# The staying power of change: sustainability of pain practice improvements after a multidimensional knowledge translation intervention

**DOI:** 10.1186/1472-6963-14-S2-P118

**Published:** 2014-07-07

**Authors:** Bonnie Stevens, Janet Yamada, Sara Promislow

**Affiliations:** 1 University of Toronto, Toronto, Canada; 2 Hospital for Sick Children, Toronto, Canada

## Background

Despite significant developments in acute pain research in hospitalized children, pain assessment and management practices for this population remain sub-optimal. To address the gap between research and practice a multidimensional, knowledge translation intervention, Evidence-based Practice for Improving Quality (EPIQ) [1] was successfully implemented at eight Canadian tertiary pediatric hospitals [2]. EPIQ was effective in improving pain practices immediately following the intervention completion in the EPIQ units compared to standard care (SC) [2]. However, the sustainability of these improvements requires consideration. The objective of this study is to determine the sustainability of the overall effect of EPIQ on pain practices 12 months post intervention.

## Materials and methods

Thirty-two inpatient care units across 8 Canadian pediatric hospitals participated in the study. Using a prospective cohort comparative design with repeated measures, EPIQ was implemented in 16 units, while 16 units continued with SC. The intervention included 4, 3-month Plan-Do-Study-Act (PDSA) cycles and was implemented over a 15 month period. Medical charts were reviewed on all 32 units at baseline (T1), intervention completion (T2), and 12 months post intervention (T3) to determine the frequency and nature of pain practices. Data were analyzed using generalized linear mixed models to identify between and within group differences for each pain practice.

## Results

There was a significant group by time interaction effect for use of any pain assessment tool (P=0.048) with the EPIQ groups demonstrating a greater change at T2 compared to the SC groups. There was a significant effect of time for use of any validated pain assessment tool (P=0.001). There was also a significant effect of time in the proportion of patients receiving a procedure-linked analgesic (P=0.034) with a significantly greater increase occurring in the EPIQ groups compared to SC groups at T2. In all significant outcomes, improvements occurred at T2 compared to baseline (T1). Pain practice changes were partially sustained at T3.

## Conclusions

Further assessment of practice changes over a longer time period (2 years) is required and a more detailed investigation of the factors that influence the sustainability of pain practice improvements, including examination of child and organization related contextual factors.
